# Effect of sex on the efficacy of patients receiving immune checkpoint inhibitors in advanced non‐small cell lung cancer

**DOI:** 10.1002/cam4.2280

**Published:** 2019-06-04

**Authors:** Chengdi Wang, Wenliang Qiao, Yuting Jiang, Min Zhu, Jun Shao, Pengwei Ren, Dan Liu, Weimin Li

**Affiliations:** ^1^ Department of Respiratory and Critical Care Medicine West China Medical School/West China Hospital, Sichuan University Chengdu China; ^2^ Lung Cancer Center West China Hospital, Sichuan University Chengdu China; ^3^ West China Medical School Sichuan University Chengdu China; ^4^ Department of Clinical Research Center for Respiratory Diseases West China Hospital, Sichuan University Chengdu China

**Keywords:** chemotherapy, CTLA‐4, immune checkpoint inhibitors, NSCLC, PD‐1, PD‐L1, sex

## Abstract

Immune checkpoint inhibitors (ICIs) have shown promising efficacy in the treatment of non‐small cell lung cancer (NSCLC). Sex‐associated dimorphism in immune system response is acknowledged, but the effect of patients’ sex on efficacy of ICIs as treatment in NSCLC still remains controversial. The present study was conducted to investigate the difference in efficacy of NSCLC patients receiving immune checkpoint inhibitors according to the sex. A total of 9583 patients involved 6567 men and 3016 women with advanced lung cancer from 15 randomized controlled trials were included in this study. An overall survival (OS) benefit of immune checkpoint inhibitors was illustrated in both male (HR 0.76, 95% CI 0.71‐0.82) and female (HR 0.73, 95% CI 0.58‐0.91) patients, and a progression‐free survival (PFS) benefit was also found in both men (HR 0.67, 95% CI 0.58‐0.77) and women (HR 0.73, 95% CI 0.56‐0.95) in NSCLC. Both PD‐1/PD‐L1 inhibitors alone and PD‐1/PD‐L1 plus chemotherapy significantly improved the OS and PFS in male patients. Whereas in females, PD‐1 inhibitors or monotherapy significantly benefited the OS but not the PFS, PD‐L1 inhibitors or combination therapy significantly prolonged the PFS but not the OS. No survival benefit was found in both male and female patients from the CTLA‐4 inhibitors. The current study indicated that the magnitude of survival benefit is sex‐dependent and male patients seemed to obtain more consistent and favorable outcomes from ICIs than women patients in NSCLC.

## 1 INTRODUCTION

Sex difference can potentially affect the immunologic responses to both self and foreign antigens.^1^ In general, women tend to trigger stronger innate and adaptive immune response compared with men, which leads to faster clearance of pathogens and greater vaccine efficacy with the lower incidence and severity of many infectious diseases caused by bacteria, fungi, viruses, and parasites.^1^, ^2^, ^3^ Whereas in the autoimmune diseases, women account for approximately 80% of all cases worldwide.^1^, ^4^ In oncology, men show an almost two‐times higher risk of mortality from many malignant cancers than women, with sex differential outcomes being greatest for bladder, esophagus, melanoma, and lung cancers.^5^, ^6^ Sex‐dependent effects probably reflect differences not only in biological factors and behavioral factors, including gene‐environment interactions and hormonal regulation, but also in the immune system.

In recent years, immune checkpoint inhibitor (ICI) targeting PD‐1/PD‐L1 (programmed cell death 1/programmed cell death 1 ligand 1) and CTLA‐4 (cytotoxic T‐lymphocyte antigen‐4) have become the most revolutionary treatment in several malignant neoplasms such as non‐small cell lung cancer (NSCLC) and melanoma.^7^ Sex hormones regulate the expression and function of PD‐1/PD‐L1 and affect the PD‐1 pathway in mediating autoimmunity.^8^, ^9^ Based on existing knowledge of published trials, male patients seemed to benefit more from immune checkpoint inhibitors. Women mount stronger immune response, which could reduce the risk of cancer mortality. A higher smoking prevalence in men increased the tumor mutational burden, which is a strong predictor of benefit from immune checkpoint inhibitors.^1^, ^10^ Conforti et al found that men had a significantly higher benefit from immune checkpoint inhibitors compared with women in all cancers especially in NSCLC (male: HR 0.72, 95% CI 0.61‐0.86; female: 0.89, 95% CI 0.71‐1.11).^11^ However, Graham et al presented that no difference in survival advantages for both men and women receiving the immune checkpoint inhibitors in patients with metastatic renal cell carcinoma.^12^ Wallis et al reported women derived greater value from the immunotherapy in NSCLC (male: HR 0.79, 95% CI 0.71‐0.88; female: 0.72, 95% CI 0.56‐0.93).^13^

The previous studies indicated that the role of sex in ICI trials in cancers were controversial. How sex associates with ICI efficacy is incompletely elucidated. To address these concerns, we perform the current study of 9583 patients to investigate the association of patient sex with the benefits from immune checkpoint inhibitors in NSCLC with recently accumulated evidence.

## 2 MATERIALS AND METHODS

### 2.1 Study design and search strategy

We searched the Embase, PubMed, and Cochrane Central Register of Controlled Trials (CENTRAL) for phase 2 and phase 3 randomized controlled trials (RCTs) from the inception to December 2018. The main search terms used in the search strategy included immune checkpoint inhibitors (anti‐programmed cell death 1 or anti‐PD‐1, anti‐programmed cell death ligand 1 or anti‐PD‐L1, anti‐cytotoxic T lymphocyte‐associated antigen 4 or anti‐CLTA‐4), specific drug names (pembrolizumab, nivolumab, atezolizumab, durvalumab, avelumab, ipilimumab, tremelimumab), randomized controlled trials, lung cancer (lung adenocarcinoma, lung squamous cell carcinoma, or NSCLC). The search strategy is provided in the supplementary materials. We also performed a computerized search of the major international conference proceedings of ESMO (European Society of Medical Oncology), ASCO (American Society of Clinical Oncology), AACR (American Association for Cancer Research), and WCLC (World Conference of Lung Cancer). Study selection was carried out in line with the PRISMA (Preferred Reporting Items for Systematic Reviews and Meta‐Analysis) guidelines.^14^

### 2.2 Study selection

To be eligible, randomized controlled trials had to assess the inhibitors of PD‐1, PD‐L1, CTLA‐4, or their combination comparing immunotherapy with other systemic treatment regimens including chemotherapy in patients with lung cancer. Studies that investigated immune checkpoint inhibitors plus chemotherapy compared with chemotherapy alone were also taken into account. Eligible studies had to have data available for the hazard ratio (HR) for overall survival (OS) or progression‐free survival (PFS) according to the sex of lung cancer. Trails that belonged to retrospective or prospective observational cohort studies or reported subgroup analysis for one sex only were excluded.

### 2.3 Data extraction and quality assessment

Two investigators independently reviewed and extracted the data including first author, publication year, study ID, trial phase, treatment, hazard ratio for overall survival or progression‐free survival stratified by patient sex with a standardized data collection form. Any discrepancies or disagreements were discussed and addressed with the consensus of all investigators. A risk of bias assessment was evaluated by using the tool recommended by the Cochrane Collaboration Handbook.^15^ Methodological quality was assessed based on: random sequence generation; allocation concealment; blinding method, assessment of outcomes; selective reporting and additional source of bias.

### 2.4 Statistical analysis

The heterogeneity was identified using Cochrane’s *Q* and *I* ^2^ statistic. For the *Q* test, *P*‐value less than 0.10 implied significant heterogeneity; for the *I* ^2 ^statistic, *I* ^2 ^value more than 50% indicated significant heterogeneity. Random effects model by DerSimonian‐Laird method was applied; otherwise the fixed effects model by Mantel‐Haenszel method was performed. We derived the hazard ratio and 95% confidence intervals (CI) for OS or PFS in the intervention/treatment group and control group for men and women patients separately. We calculated the pooled HR of OS or PFS in male and female patients applying random effects model or fixed effects model. We also did subgroup analyses to investigate the variation of effect of sex immunotherapy efficacy. Egger’s test and Begg’s test were used to evaluate potential publication bias. All analysis was performed with Stata version 15.0 (StataCorp, College Station, TX). All reported *P* values were two‐sided and *P*‐value less than 0.05 was used to indicate statistical significance.

## 3 RESULT

### 3.1 Literature search

A total of 2784 potentially related articles were identified from online database by the initial search strategy. After eligibility screening the abstracts and reviewing the full texts, 15 randomized controlled trials (RCTs) involving 9583 patients were finally included in the present study (Figure S1).^16^, ^17^, ^18^, ^19^, ^20^, ^21^, ^22^, ^23^, ^24^, ^25^, ^26^, ^27^, ^28^, ^29^, ^30^ Data from all eligible trials were obtained from published articles and conference proceedings (KEYNOTE 042, IMpower131 and IMpower132).

### 3.2 Study characteristics

The main characteristics of the included 15 randomized controlled trials were summarized in Table 1, of which 6567 were male and 3016 were female. Seven RCTs reported data on both OS and PFS, five RCTs with only OS data, and three RCTs with only PFS data. All these trials with one phase 2 trail, 14 phase 3 trials were international, multicenter studies published in the past 4 years. We found seven randomized controlled trials with PD‐1 inhibitors (pembrolizumab and nivolumab), six trials with PD‐L1 inhibitors (atezolizumab, durvalumab, avelumab), one trial with CTLA‐4 inhibitor (ipilimumab), and one trial with PD‐1 inhibitor plus CTLA‐4 inhibitor (nivolumab & ipilimumab).

**Table 1:** Characteristics and outcomes data of included randomized controlled trials

First Author	Year	Study ID	Trial	Cancer Target	Intervention/Treatment (No.)	No of Patients male/female	OS for Sex Men/Women	PFS for Sex Men/Women
Hellmann	2018	CheckMate 227	3	NSCLC	Nivolumab + Ipilimumab (139)	M: 204	M: NA	M: 0.52(0.36‐0.74)
				PD‐1 + CTLA‐4	Chem (160)	F: 95	F: NA	F: 0.70(0.41‐1.20)
Jotte	2018	IMpower 131	3	NSCLC	Atezolizumab + Chem (343)	M: 557	M: NA	M: 0.71(0.59‐0.85)
				PD‐L1	Chem (340)	F: 126	F: NA	F: 0.66(0.45‐0.97)
Papadimitrakopoulou	2018	IMpower 132	3	NSCLC	Atezolizumab + Chem (292)	M: 384	M: NA	M: 0.64(0.51‐0.79)
				PD‐L1	Chem (286)	F: 194	F: NA	F: 0.51(0.36‐0.71)
Barlesi	2018	JAVELIN Lung 200	3	NSCLC	Avelumab (396)	M: 367	M: 0.83(0.64‐1.08)	M: NA
				PD‐L1	Chem (396)	F: 162	F: 1.08(0.74‐1.59)	F: NA
Lopes	2018	KEYNOTE 042	2	NSCLC	Nivolumab (637)	M: 902	M: 0.80(0.68‐0.94)	M: NA
				PD‐1	Chem (637)	F: 372	F: 0.89(0.68‐1.17)	F: NA
Gandhi	2018	KEYNOTE 189	3	NSCLC	Pembrolizumab + Chem (410)	M: 363	M: 0.70(0.50‐0.99)	M: 0.66(0.50‐0.87)
				PD‐1	Chem (206)	F: 253	F: 0.29(0.19‐0.44)	F: 0.40(0.29‐0.54)
Paz‐Ares	2018	KEYNOTE 407	3	NSCLC	Pembrolizumab + Chem (278)	M: 455	M: 0.69(0.51‐0.94)	NA
				PD‐1	Chem (281)	F: 104	F: 0.42(0.22‐0.81)	NA
Antonia	2018	PACAFIC	3	NSCLC	Durvalumab plus Chemoradiotherapy (476)	M: 500	M: 0.78(0.59‐1.03)	M: 0.54(0.41‐0.71)
				PD‐L1	Chemoradiotherapy (237)	F: 213	F: 0.46(0.30‐0.73)	F: 0.54(0.37‐0.79)
Govindan	2017	CA184‐104	3	NSCLC	Ipilimumab + Chem (388)	M: 635	M: 0.85(0.71‐1.02)	M: NA
				CTLA‐4	Chem (361)	F: 114	F: 1.33(0.84‐2.11)	F: NA
Carbone	2017	CheckMate 026	3	NSCLC	Nivolumab (271)	M: 332	M: 0.97(0.74‐1.26)	M: 1.05(0.81‐1.37)
				PD‐1	Chem (270)	F: 209	F: 1.15(0.79‐1.66)	F: 1.36(0.98‐1.90)
Rittmeyer	2017	OAK	3	NSCLC	Atezolizumab (425)	M: 520	M: 0.79(0.64‐0.97)	M: NA
				PD‐L1	Docetaxel (425)	F: 330	F: 0.64(0.49‐0.85)	F: NA
Herbst	2016	KEYNOTE 010	2/3	NSCLC	Pembrolizumab (691)	M: 634	M: 0.65(0.52‐0.81)	M: 0.78(0.64‐0.94)
				PD‐1	Chem (343)	F: 399	F: 0.69(0.51‐0.94)	F: 1.02(0.78‐1.32)
Reck	2016	KEYNOTE 024	3	NSCLC	Pembrolizumab (154)	M: 187	M: 0.54(0.36‐0.80)	M: 0.39(0.26‐0.58)
				PD‐1	Chem (151)	F: 118	F: 0.96(0.56‐1.64)	F: 0.75(0.46‐1.21)
Borghaei	2015	CheckMate 057	3	NSCLC	Nivolumab (292)	M: 319	M: 0.73(0.56‐0.96)	M: 0.81(0.63‐0.96)
				PD‐1	Chem (290)	F: 263	F: 0.78(0.58‐1.04)	F: 1.04(0.80‐1.37)
Brahmer	2015	CheckMate 017	3	NSCLC	Nivolumab (135)	M: 208	M: 0.57(0.41‐0.78)	M: 0.63(0.46‐0.85)
				PD‐1	Chem (137)	F: 64	F: 0.67(0.36‐1.25)	F: 0.71(0.40‐1.26)

Abbreviations: Chem: chemotherapy; CI: confidence interval; CTLA4: cytotoxic T lymphocyte associated antigen 4; F: female; HR: hazard ratio; ICI: immune checkpoint inhibitor; M: male; NA: not available; NSCLC: non‐small‐cell lung cancer; OS: overall survival; PD‐1: Programmed cell death 1; PD‐L1: Programmed cell death 1 ligand 1; PFS: progression‐free survival.

Several studies may warrant further explanation due to the unique designs. The KEYNOTE 010 study tested two different doses of pembrolizumab (2 mg/kg and 10 mg/kg) vs docetaxel in advanced NSCLC patients. In this scenario, the pooled HR for OS and PFS was considered. CheckMate 227 trial was designed to evaluate different nivolumab‐based regimens (nivolumab monotherapy, nivolumab plus chemotherapy, nivolumab plus ipilimumab) versus chemotherapy in distinct patient populations. The part of CheckMate 227 trial focusing on nivolumab plus ipilimumab versus chemotherapy among patients with NSCLC was identified due to available data.

### 3.3 Effect of sex on overall survival

**Figure 1:**
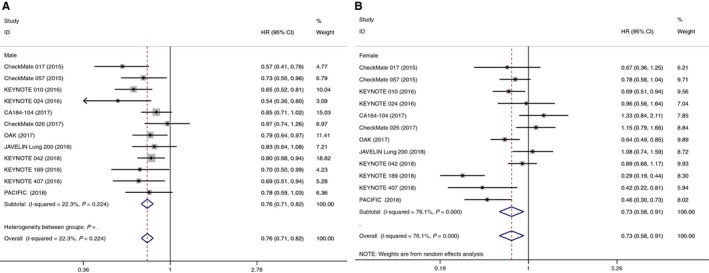
Forest plot of hazard ratios comparing overall survival in patients who received immune checkpoint inhibitors with control treatment in male (A) and female (B)

Twelve RCTs provided the overall survival data in terms of sex. The pooled result demonstrated that patients receiving immune checkpoint inhibitors (PD‐1, PD‐L1, or CTLA‐4 inhibitors) had a significantly reduced risk of death for both men (HR 0.76, 95% CI 0.71‐0.82, *P *＜ 0.001) and women (HR 0.73, 95% CI 0.58‐0.91, *P* = 0.007) (Figure 1). There was substantial between‐study heterogeneity in female patients (*I* ^2^ = 76.1%, *P *＜ 0.001), but not in male patients (*I* ^2^ = 22.3%, *P* = 0.224). In subgroup analysis of male patients, there was an OS benefit in the anti‐PD‐1 treatment (HR 0.73, 95% CI 0.67‐0.80) and anti‐PD‐L1 treatment (HR 0.80, 95% CI 0.69‐0.92). Whereas in female patients, OS benefit was found in the anti‐PD‐1 treatment (HR 0.69, 95% CI 0.52‐0.93) but not in anti‐PD‐L1 treatment (HR 0.69, 95% CI 0.44‐1.07). Monotherapy (ICI alone) seems to have an OS benefit compared with combination therapy (ICI inhibitors combination or ICI plus chemotherapy) for both men and women. No survival benefit was found in both male and female patients from the CTLA‐4 inhibitors (Table 2).

**Table 2:** Subgroup‐analysis of included RCTs for overall survival and progression‐free survival in Men and Women

OS					PFS				
Variable	No. of Trials	No. of patients	Pooled HR (95% CI)		Variable	No. of trials	No. of patients	Pooled HR (95% CI)	
		Men/Women	Men	Women			Men/Women	Men	Women
ICI drug					ICI drug				
Anti‐PD‐1	8	3400/1782	0.73 (0.67‐0.80)	0.69 (0.52‐0.93)	Anti‐PD‐1	6	2043/1306	0.71 (0.58‐0.88)	0.83 (0.57‐1.20)
Anti‐PD‐L1	3	1387/705	0.80 (0.69‐0.92)	0.69 (0.44‐1.07)	Anti‐PD‐L1	3	1441/533	0.64 (0.56‐0.74)	0.56 (0.45‐0.69)
Anti‐CTLA‐4	1	635/114	0.85 (0.71‐1.02)	1.33 (0.84‐2.11)	Anti‐CTLA‐4	‐	‐	‐	‐
Study methodology					Study methodology				
Monotherapy	8	3469/1917	0.75 (0.69‐0.82)	0.83 (0.71‐0.96)	Monotherapy	5	1680/1053	0.72 (0.56‐0.92)	1.02 (0.84‐1.23)
Combination therapy	4	1953/684	0.78 (0.69‐0.89)	0.52 (0.26‐1.04)	Combination therapy	5	2008/881	0.64 (0.57‐0.71)	0.53 (0.43‐0.64)

Abbreviations: Combination therapy, ICI inhibitors combination or ICI plus chemotherapy; Monotherapy, immune checkpoint inhibitor (ICI) alone.

### 3.4 Effect of sex on progression‐free survival

**Figure 2:**
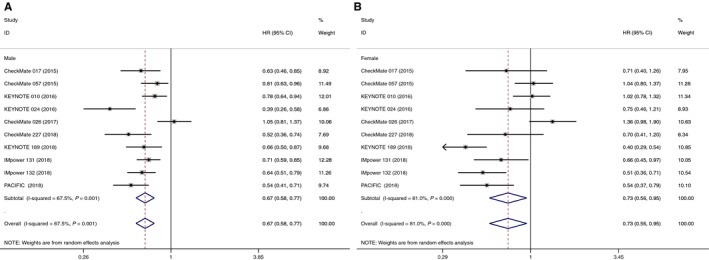
Forest plot of hazard ratios comparing progression‐free survival in patients who received immune checkpoint inhibitors with control treatment in male (A) and female (B)

The current study of PFS was based on 10 RCTs providing the required data. A statistically significant PFS benefit was found in both men (HR 0.67, 95% CI 0.58‐0.77) and women (HR 0.73, 95% CI 0.56‐0.95) who received immune checkpoint inhibitors (compared with available standard therapies) (Figure 2). Heterogeneity was observed among both men and women. Subgroup analyses were also performed. Immunotherapy led to statistically longer PFS across all tested subgroups of anti‐PD‐1 drug (HR 0.71, 95% CI 0.58‐0.88) and anti‐PD‐L1 drug (HR 0.64, 95% CI 0.56‐0.74), monotherapy (HR 0.72, 95% CI 0.56‐0.92) and combination therapy (HR 0.64, 95% CI 0.57‐0.71) in male NSCLC patients. Whereas in female patients, PFS benefit was observed in the anti‐PD‐L1 treatment (HR 0.56, 95% CI 0.45‐0.69) and combination therapy (HR 0.53, 95% CI 0.43‐0.64), but not in anti‐PD‐1 treatment (HR 0.83, 95% CI 0.57‐1.20) and monotherapy (HR 1.02, 95% CI 0.84‐1.23) (Table 2).

### 3.5 Risk of bias and publication bias and sensitivity analysis

Results from the risk of bias of the included RCTs are shown in Table S1. All studies were well designed and reported at low risk of sequence generation, incomplete outcome data and selective reporting. Several studies were at risk of binding and allocation concealment. The other main source of risk of bias was that the data in three trials (IMpower 131, IMpower 132, and KEYNOTE 042) were from the conference presentations. Both of which were unlikely to have the influence on the result. The plots suggested the absence of publication bias and sensitivity analysis indicated that the outcome remained consistent (Figures S2 and S3).

## 4 DISCUSSION

Sex‐based differences of the immunologic responses may reflect the different outcome in the efficacy of immune checkpoint inhibitors in male and female. The aim of the current study is to analyse differences in the response to immunotherapy in both sexes. We investigate OS data from 12 RCTs with 8023 patients and PFS data from 10 RCTs with 5622 patients. The result of the current study demonstrated that immune checkpoint inhibitors could improve overall survival and progression‐free survival for patients of both sexes in non‐small cell lung cancer. Interestingly, this study indicated a greater net value of immune checkpoint inhibitors for women (HR 0.73, 95% CI 0.58‐0.91) than for men (HR 0.76, 95% CI 0.71‐0.82) in terms of overall survival. One possible explanation is that the stronger immune responses from women might lower the risk of cancer mortality. This result seems consistent with Wallis's study (male: HR 0.79, 95% CI 0.71‐0.88; female: 0.72, 95% CI 0.56‐0.93) which suggested a greater immunotherapy advantage in women, but contrary to Conforti's study (male: HR 0.72, 95% CI 0.61‐0.86; female: 0.89, 95% CI 0.71‐1.11) that indicated a greater immunotherapy benefit in men in NSCLC.^11^, ^13^ However, interestingly, for progression‐free survival, men patients have a stronger advantage from immunotherapy compared with women patients (male: HR 0.67, 95% CI 0.58‐0.77; female: HR 0.73, 95% CI 0.56‐0.95). This magnitude of PFS benefit seems clinically relevant. The previous study indicated that men treated with PD‐1 inhibitor had two‐times lower the risk of death than women.^31^ The result was partly in accordance with Grassadonia’s study (male: HR 0.67, 95% CI 0.55‐0.80; female: HR 0.77, 95% CI 0.57‐1.05) and Wu’s study (male: HR 0.60, 95% CI 0.36‐0.84; female: HR 0.88, 95% CI 0.68‐1.08), which revealed no significant improvement in PFS in women in NSCLC.^32^, ^33^ Therefore, it remains a controversy and under‐investigated issue whether magnitude of overall survival and progression‐free survival advantage from immunotherapy are sex‐dependent.

Five different meta‐analysis have been reported to investigate the efficacy of immune checkpoint inhibitors according to the sex of cancer patients.^11^, ^13^, ^32^, ^33^, ^34^ In the previous meta‐analysis conducted by Botticelli and colleagues,^34^ there was no significant improvement of overall survival in male cancer patients treated with anti‐PD‐1 or anti‐CTLA‐4 antibodies. No available data of OS data and PFS data was found in the stratified analysis of cancer type like NSCLC according to the sex. In the meta‐analysis of Wu and colleagues,^33^ subgroup analysis by type of cancer, four trails of NSCLC including 2192 patients showed that men had the better survival benefit compared with women. Grassadonia et al reported that male patients had both the better OS advantage from six RCTs and PFS advantage from eight RCTs than female patients in NSCLC.^32^ In the study of only included OS analysis performed by Conforti and colleagues, the pooled overall survival HR was lower from six RCTs of 3482 patients in male patients than female patients.^11^ Recently, Wallis et al found in the subgroup analysis of 11 RCTs including 4520 men and 2125 women that female had the better OS benefit from immunotherapy compared to male in NSCLC.^13^ Small sample sizes may lead to an increased false discovery rate or even false‐positive outcomes. The current study included an updated search of the largest number of patients (9583 patients) from 15 RCTs with relatively complete immunotherapy agents, of which increased the credibility of our analysis.

It still remains unknown which is better efficacy of PD‐1, PD‐L1, or CTLA‐4 inhibitors for men and women. A previous meta‐analysis emphasized that PD‐1 inhibitors for men would consequently have better OS benefit than women.^33^ Our result indicated that women had a lower HR of OS meaning a higher magnitude of overall survival efficacy, but no significant PFS benefit from PD‐1 inhibitors. A preclinical study hypothesized that PD‐L1 inhibitors for women would become more efficacious consequently compared to men.^35^ Our result showed that women received PFS benefit but no OS advantage from PD‐L1 inhibitors. For men patients in NSCLC, both PD‐1 inhibitors and PD‐L1 inhibitors could improve the survival of OS and PFS. From this point of view, there was a significantly higher magnitude of anti‐PD‐1 and anti‐PD‐L1 efficacy in men. Grassadonia et al demonstrated that CTLA‐4 inhibitors tended to improve the survival in male patients but not in female patients.^32^ Our study revealed that both male and female patients had no survival benefit from anti‐CTLA‐4 treatment. The intriguing scenario is that why survival improvement of CTLA‐4 inhibitors is not consistent with PD‐1/ PD‐L1 inhibitors. One possible explanation is that their mechanisms are different. CTLA‐4 is mainly expressed on T lymphocytes and PD‐1/ PD‐L1 is pervasively expressed on B cells, T cells, myeloid cells, and non‐lymphoid organs.^36^, ^37^ The CTLA‐4 inhibitors can reactivate suppressed T lymphocytes and trigger anti‐tumor response. PD‐1/ PD‐L1 inhibitors can lead to peripheral T‐cell proliferation and infiltration into the tumor, inducing an objective anti‐tumor response. Therefore, further studies with functional analysis are warranted to investigate the issue.

Preclinical studies showed that conventional therapy including the radiotherapy and chemotherapy could kill tumor cells and induce PD‐L1 expression on tumor cells.^38^, ^39^ Fiorica and colleagues reported that immune checkpoint inhibitors plus radiotherapy gained both OS and PFS benefit with tolerated toxicities in patients of NSCLC.^40^ Several studies demonstrated that immune checkpoint inhibitors alone or plus chemotherapy could improve tumor response and prolong survival in both male and female patients in NSCLC.^21^, ^22^, ^26^ However, other studies showed that immune checkpoint inhibitors alone or plus chemotherapy had no obvious survival benefit in both men and women in NSCLC.^24^, ^25^ Our study indicated that male patients could obtain OS and PFS benefit from both monotherapy and combination therapy in NSCLC. Whereas in female patients, monotherapy could prolong OS but not PFS and combination therapy could prolong PFS but not OS. From all the subgroups, we could conclude that efficacy of immune checkpoint inhibitors may not be equally effective according to sex and ICI may seem more beneficial in men of NSCLC patients.

To the best of our knowledge, we speculate on three possible reasons that could explain the difference between men and women. First, sex dimorphism in immunity indicates that the stronger immune responses occurring in women could lower the risk of cancer mortality and higher incidence of autoimmune diseases also could make them more susceptible to produce ICI‐related adverse events causing higher possibility of treatment discontinuation. Second, gender dimorphism in behaviors such as higher smoking frequency in men, which increases tumor mutational burden (TMB) greatly and such correlates with immune checkpoint inhibitor efficacy. Third, lower smoking prevalence with lower tumor immunogenic landscape and higher EGFR mutation might lead to these results among women patients with lung cancer, and those female patients with EGFR tumor mutations receive no survival benefit from the immune checkpoint inhibitors. We performed a systematic review and meta‐analysis to investigate the efficacy of immunotherapy in lung cancer according to sex and demonstrated that male patients could derive larger relative survival benefit from immune checkpoint inhibitors (compared with available standard therapies) then female patients.

The present study had several limitations. First, this study relied on published randomized controlled trial subgroup HRs of OS and PFS rather than individual participant data. Second, several RCTs of immune checkpoint inhibitors excluded due to lack of available sex‐subgroup analysis data that might indicate sex differences if included in the analysis. Third, the OS and PFS data from all included RCTs were not mature enough and update studies will provide final survival data. Fourth, differences from results between both male and female patients might be attributed to other factors such as prevalence of autoimmune diseases, difference in the number of both men and women, and difference of life style. The above limitations may have a certain influence on the final outcome. Despite the limitations, the current study is the largest meta‐analysis incorporating outcomes from 15 RCTs involved over 9000 patients in NSCLC, providing the increased credibility of results.

## 5 CONCLUSIONS

In the current study, immune checkpoint inhibitors are significantly related with prolonged overall survival and prolonged progression‐free survival in both male and female patients in NSCLC. Both PD‐1/PD‐L1 inhibitors alone and PD‐1/PD‐L1 plus chemotherapy significantly improved the OS and PFS in male patients. Whereas in females, PD‐1 inhibitors or monotherapy significantly benefited the OS but not the PFS, PD‐L1 inhibitors or combination therapy significantly prolonged the PFS but not the OS. No survival benefit was found in both male and female patients from the CTLA‐4 inhibitors. The current study indicated that the magnitude of survival benefit is sex‐dependent and male patients seemed to obtain more consistent and favorable outcomes from ICIs than women patients in NSCLC. Prospective randomized controlled trials of greater inclusion of women stratified by sex and deeper understanding of molecular mechanisms of tumor immune response and response are needed to validate the difference of efficacy of immunotherapy in male and female patients in NSCLC.

## CONFLICTS OF INTEREST

The authors declare no conflict of interests.

## AUTHORS’ CONTRIBUTIONS

Weimin Li and Dan Liu contributed to conceptualization and supervision. Chengdi Wang, Wenliang Qiao, Yuting Jiang, Pengwei Ren contributed to data acquisition, data interpretation and statistical analysis. Chengdi Wang, Min Zhu and Jun Shao wrote and reviewed the manuscript. All the authors approved the final version.
