# Supplementary figures and images for: Effect of sex on the efficacy of patients receiving immune checkpoint inhibitors in advanced non‐small cell lung cancer
Source: Cancer Med. 2019 Jun 4;8(8):4023–31. doi: 10.1002/cam4.2280 (PMC6639192; doi:10.1002/cam4.2280)

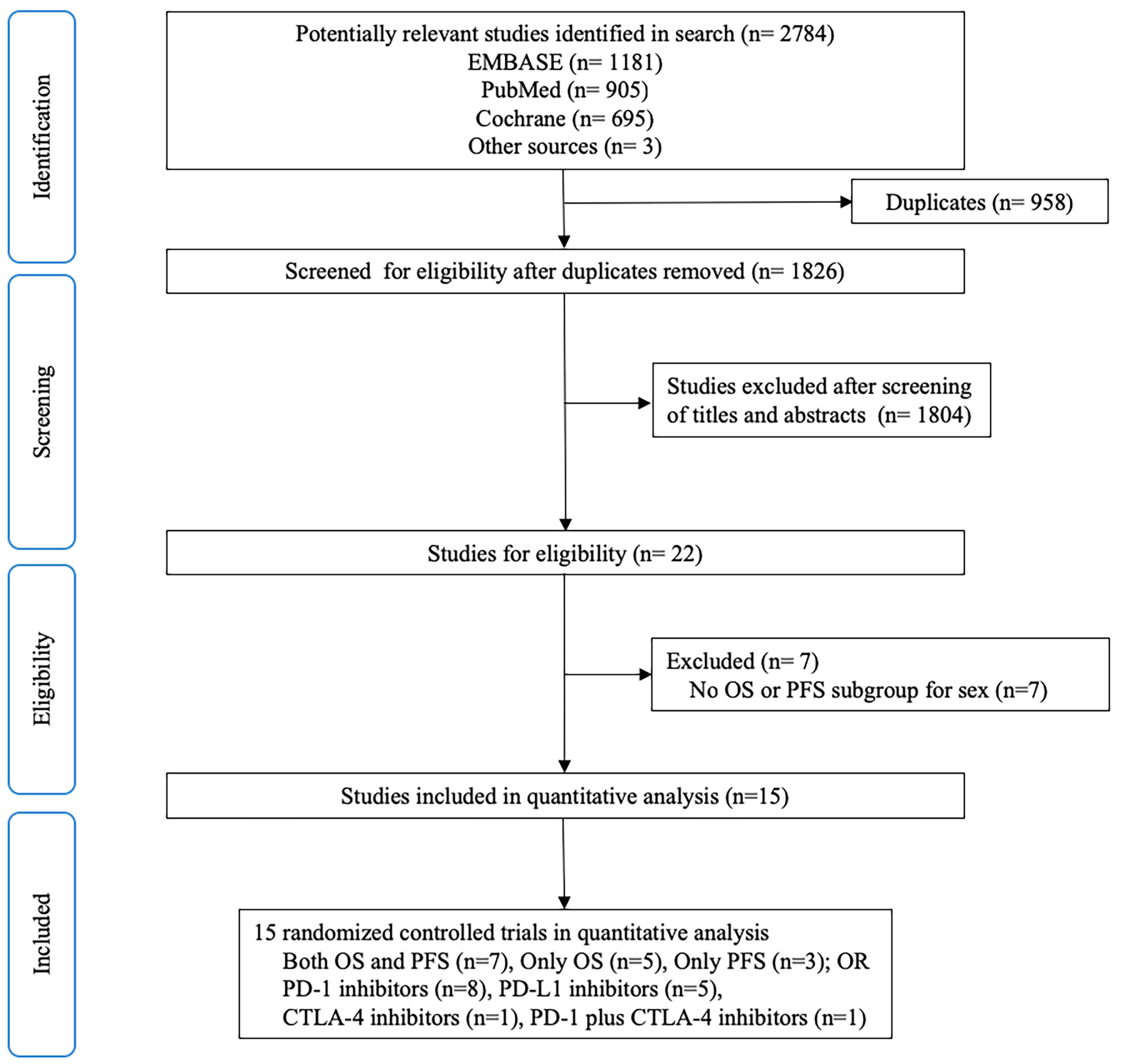

Supplement: Supplementary file 1 [file CAM4-8-4023-s001.tif]

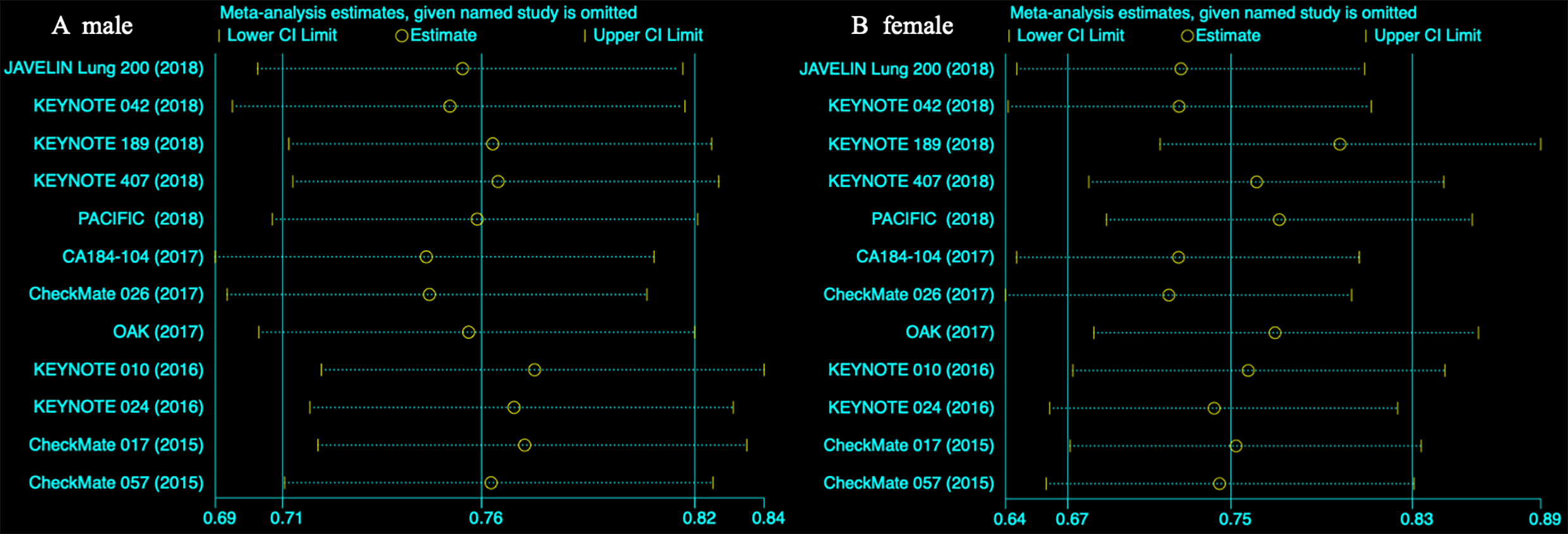

Supplement: Supplementary file 2 [file CAM4-8-4023-s002.tif]

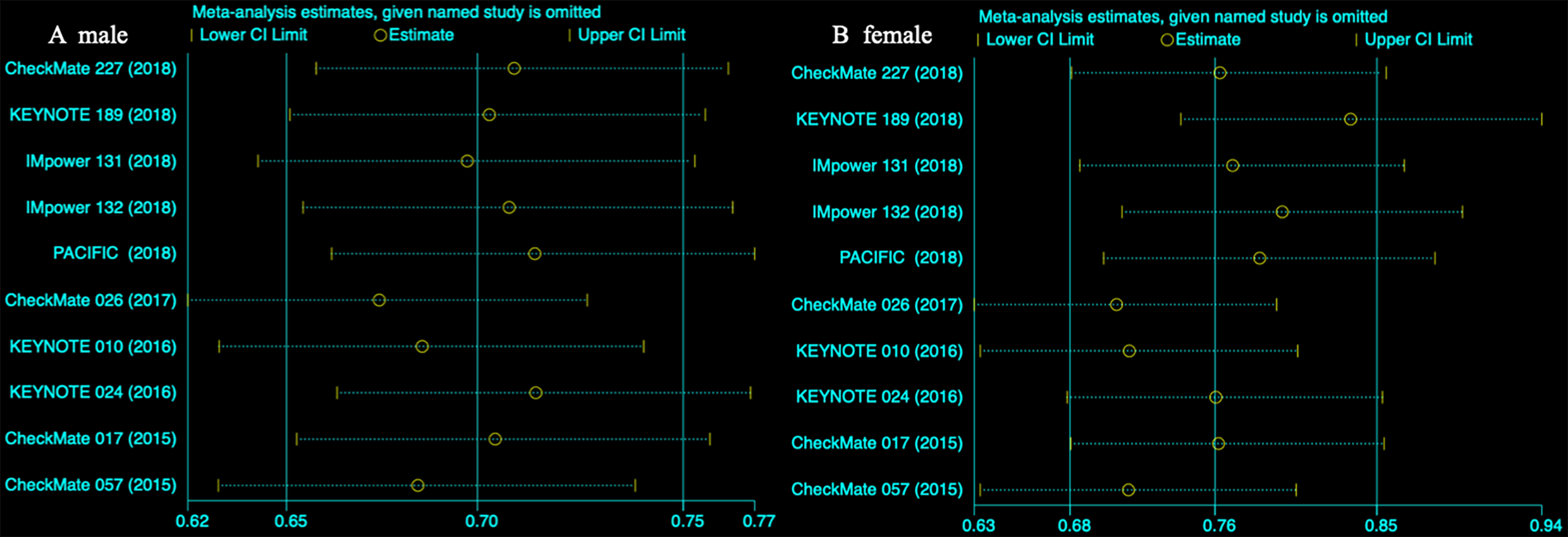

Supplement: Supplementary file 3 [file CAM4-8-4023-s003.tif]
