# Supplementary material for: Effect of sex on the efficacy of patients receiving immune checkpoint inhibitors in advanced non‐small cell lung cancer
Source: Cancer Med. 2019 Jun 4;8(8):4023–31. doi: 10.1002/cam4.2280 (PMC6639192; doi:10.1002/cam4.2280)
Supplement: Supplementary file 4 [file CAM4-8-4023-s004.docx]

**Supplementary Materials**

**Search strategy**

**PubMed**: 905 results

((“Nivolumab "[All Fields] OR "Opdivo"[All Fields] OR "ONO-4538"[All Fields] OR "MDX-1106"[All Fields] OR "BMS-936558"[All Fields] OR "Nivo"[All Fields] OR "Pembrolizumab"[Supplementary Concept] OR "Pembrolizumab"[All Fields] OR "lambrolizumab"[All Fields] OR "keytruda"[All Fields] OR "SCH 900475"[All Fields] OR "MK-3475"[All Fields]) OR "Nivolumab"[Supplementary Concept])) OR (("Avelumab"[Supplementary Concept] OR " Avelumab "[All Fields] OR "Atezolizumab"[Supplementary Concept] OR "Atezolizumab "[All Fields] OR "MSB0010718C"[All Fields] OR "Tecentriq"[All Fields] OR "RO5541267"[All Fields] OR "RG7446"[All Fields] OR "MPDL3280A"[All Fields])) OR ("Durvalumab"[Supplementary Concept] OR "Durvalumab "[All Fields] OR "MEDI-4736"[All Fields] OR "MEDI4736"[All Fields])) OR)) OR ("Ipilimumab"[Supplementary Concept] OR "Ipilimumab"[All Fields] OR "MDX-CTLA-4"[All Fields] OR "Yervoy"[All Fields])) OR ("Tremelimumab"[Supplementary Concept] OR " Tremelimumab"[All Fields])) OR ("programmed cell death-1"[All Fields] OR "PD-1 "[All Fields] OR "programmed cell death ligand-1"[All Fields] OR "PD-L1"[All Fields] OR "cytotoxic T-lymphocyte associated antigen-4"[All Fields] OR "CTLA-4"[All Fields] OR "immune checkpoint inhibitor"[All Field]))AND ("lung carcinoma"[ All Fields] OR "pulmonary carcinoma"[ All Fields] OR ("lung cancer"[All Fields] OR "lung tumor"[All Fields] OR "NSCLC"[Title/Abstract])) AND ("clinical trials as topic"[MeSH Terms] OR "trial"[Title/Abstract] OR "study"[Title/Abstract] OR "randomized controlled trial"[Title/Abstract]))

**Embase**:1,181 results

('Nivolumab' OR 'Opdivo' OR 'ONO-4538' OR 'MDX-1106' OR 'BMS-936558' OR 'Nivo' OR 'Pembrolizumab' OR 'lambrolizumab' OR 'keytruda' OR 'SCH 900475' OR 'MK- 3475' OR 'Atezolizumab' OR 'Tecentriq' OR 'Durvalumab' OR 'MEDI-4736' OR 'MEDI4736' OR 'Avelumab' OR ' Ipilimumab' OR 'MDX-CTLA-4' OR ' Yervoy' OR 'Tremelimumab' OR 'immiune checkpoint inhibitor' OR 'programmed cell death-1' OR 'PD-1' OR ' programmed cell death ligand-1' OR 'PD-L1' OR 'CTLA-4') AND ('lung cancer' OR 'lung tumor' OR 'NSCLC' OR 'lung carcinoma' OR 'pulmonary carcinoma') AND ('randomized controlled trial' or 'controlled clinical trial') AND ‘human’

**Cochrane**: 695 results

('Pembrolizumab' OR 'lambrolizumab' OR 'keytruda' OR 'SCH 900475' OR 'MK- 3475' OR 'Nivolumab' OR 'Opdivo' OR 'ONO-4538' OR 'MDX-1106' OR 'BMS-936558' OR 'Nivo' OR ' Atezolizumab' OR 'MSB0010718C' OR 'Tecentriq' OR 'RO5541267' OR 'RG7446' OR 'MPDL3280A' OR 'Durvalumab' OR 'MEDI-4736' OR 'MEDI4736' OR 'Avelumab' OR ' Ipilimumab ' OR 'Tremelimumab' OR 'checkpoint inhibitor' OR 'programmed cell death-1' OR 'PD-1' OR ' programmed cell death ligand-1' OR 'PD-L1' OR 'CTLA-4') AND ('lung carcinoma' OR 'pulmonary carcinoma' OR 'lung cancer' OR 'lung tumor' OR 'NSCLC' ) AND ( 'trials')

Table S1. Quality assessment: risk of bias by Cochrane Collaboration’s tool

| **Study ID** | **Year** | **Sequence generation** | **Allocation concealment** | **Blinding** | **Incomplete outcome data** | **Selective reporting** | **Other source of bias** |
| --- | --- | --- | --- | --- | --- | --- | --- |
| Checkmate 227 | 2018 | Low Risk | Low Risk | Low Risk | Low Risk | Low Risk |  |
| IMpower 131 | 2018 | Low Risk | Low Risk | Low Risk | Low Risk | Low Risk | Data from the abstract and the presentation slides |
| IMpower 132 | 2018 | Low Risk | Low Risk | Low Risk | Low Risk | Low Risk | Data from the abstract and the presentation slides |
| JAVELIN Lung 200 | 2018 | Low Risk | At Risk | At Risk | Low Risk | Low Risk |  |
| KEYNOTE 042 | 2018 | Low Risk | At Risk | At Risk | Low Risk | Low Risk | Data from the abstract and the presentation slides |
| KEYNOTE 189 | 2018 | Low Risk | Low Risk | Low Risk | Low Risk | Low Risk |  |
| KEYNOTE 407 | 2018 | Low Risk | Low Risk | Low Risk | Low Risk | Low Risk |  |
| PACAFIC | 2018 | Low Risk | Low Risk | Low Risk | Low Risk | Low Risk |  |
| CA184-104 | 2017 | Low Risk | At Risk | Low Risk | Low Risk | Low Risk |  |
| CheckMate 026 | 2017 | Low Risk | At Risk | At Risk | Low Risk | Low Risk |  |
| OAK | 2017 | Low Risk | At Risk | At Risk | Low Risk | Low Risk |  |
| KEYNOTE 010 | 2016 | Low Risk | At Risk | At Risk | Low Risk | Low Risk |  |
| KEYNOTE 024 | 2016 | Low Risk | At Risk | At Risk | Low Risk | Low Risk |  |
| CheckMate 057 | 2015 | Low Risk | At Risk | At Risk | Low Risk | Low Risk |  |
| CheckMate 017 | 2015 | Low Risk | At Risk | At Risk | Low Risk | Low Risk |  |


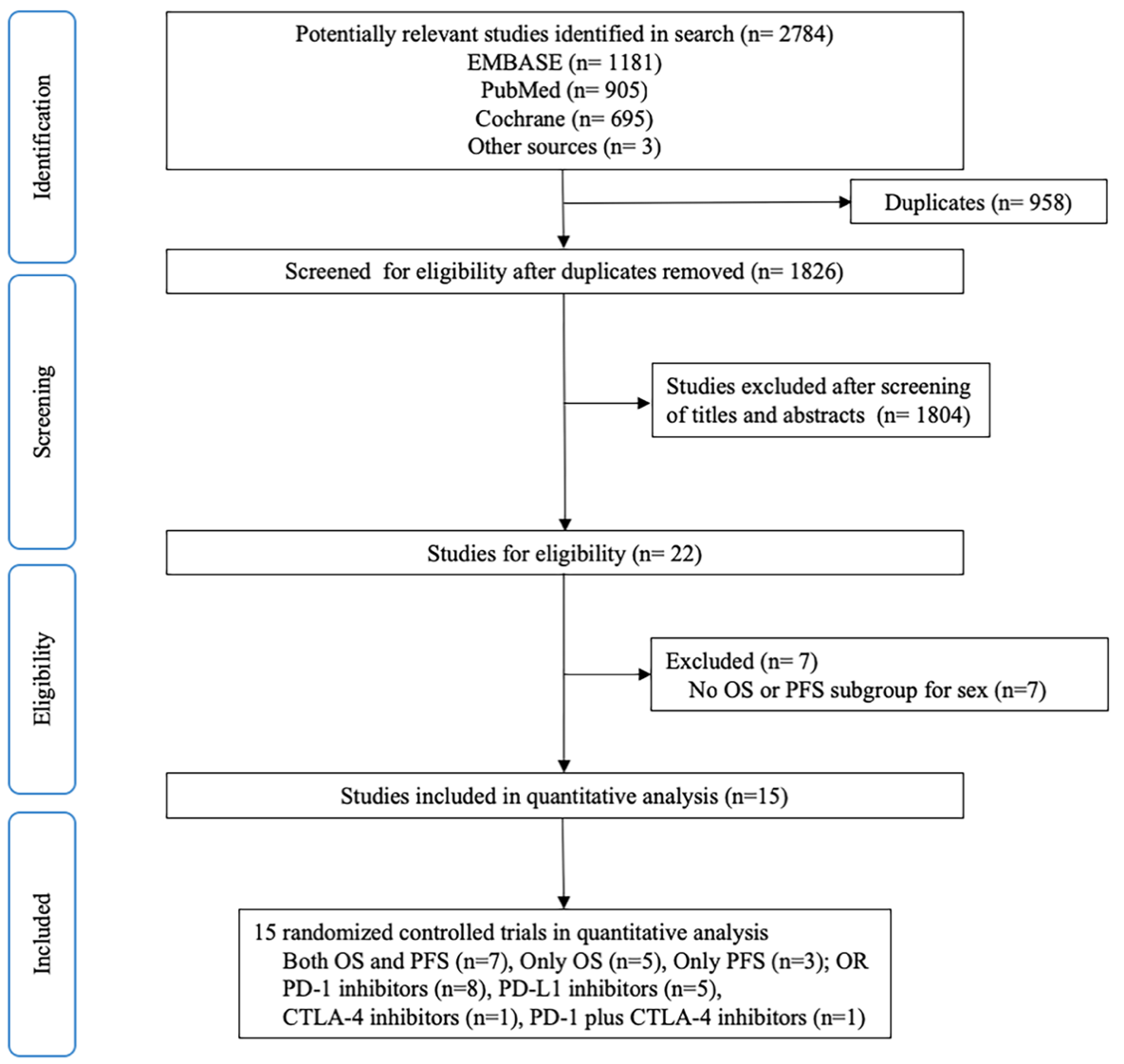


Figure S1: Study selection procedure


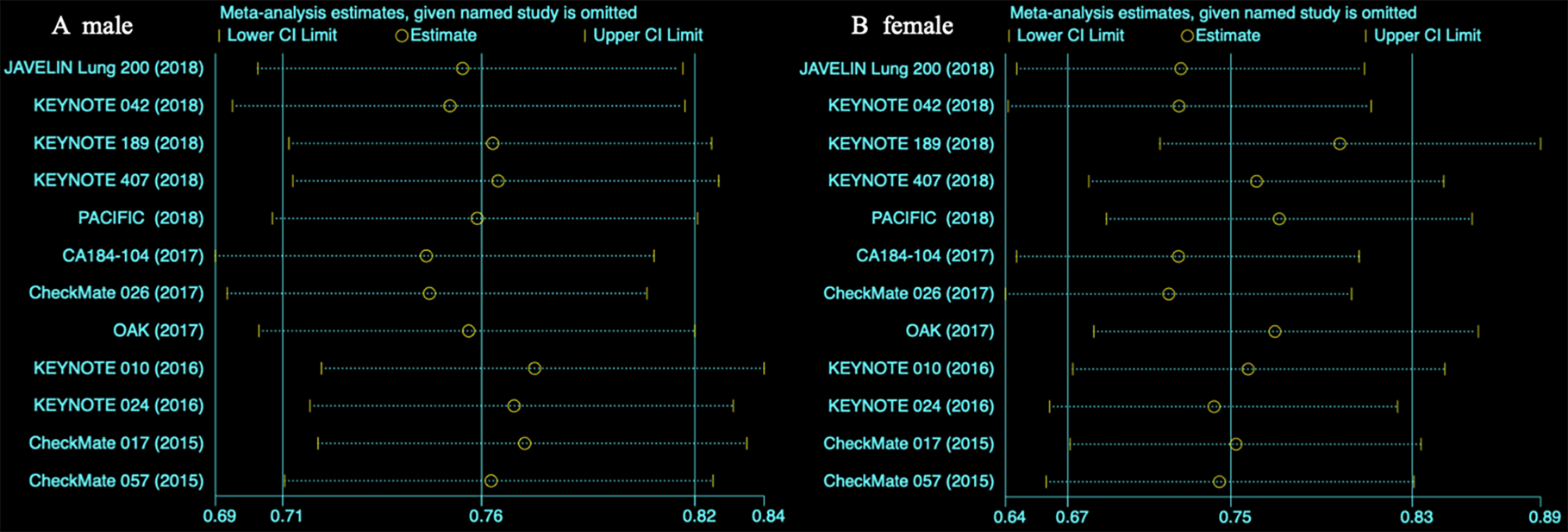


Figure S2: Sensitivity analyses of overall survival by repeating the pooled analyses with one study omitted at a time


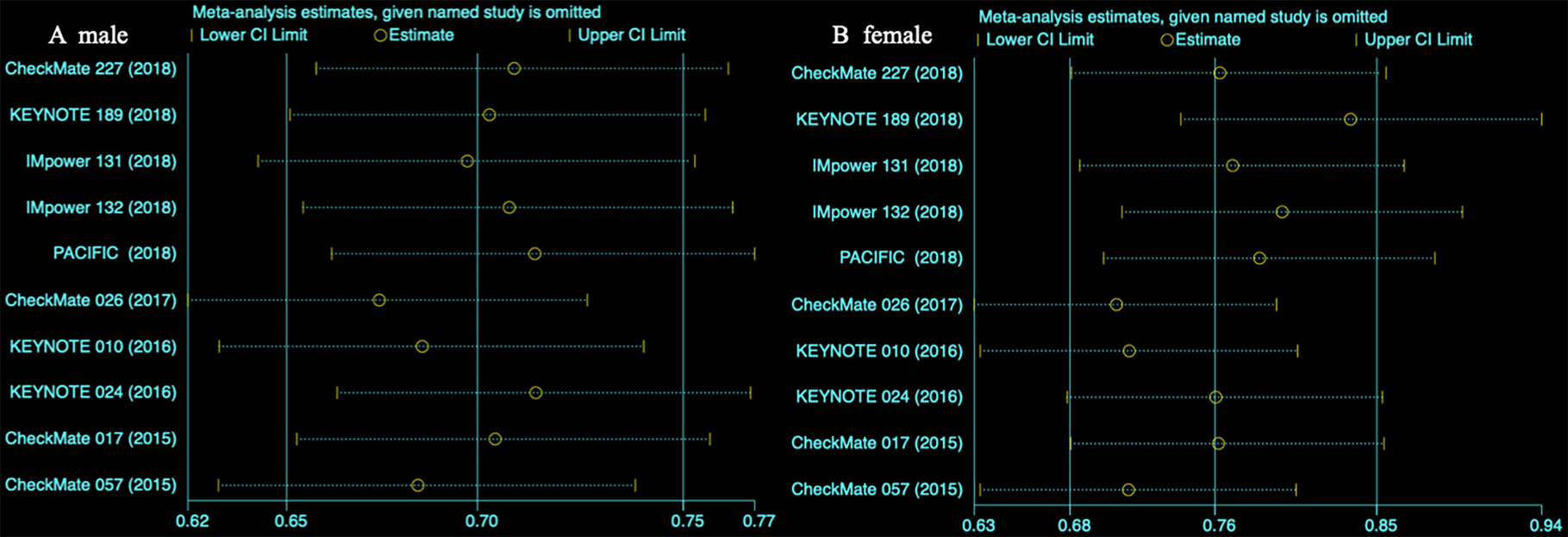


Figure S3: Sensitivity analyses of progression-free survival by repeating the pooled analyses with one study omitted at a time
